# *Mab21l2* Is Essential for Embryonic Heart and Liver Development

**DOI:** 10.1371/journal.pone.0032991

**Published:** 2012-03-08

**Authors:** Yohei Saito, Takuya Kojima, Naoki Takahashi

**Affiliations:** Department of Applied Biological Chemistry, Graduate School of Agricultural and Life Sciences, The University of Tokyo, Bunkyo-ku, Tokyo, Japan; Brigham & Women's Hospital - Harvard Medical School, United States of America

## Abstract

During mouse embryogenesis, proper formation of the heart and liver is especially important and is crucial for embryonic viability. In this study, we showed that *Mab21l2* was expressed in the trabecular and compact myocardium, and that deletion of *Mab21l2* resulted in a reduction of the trabecular myocardium and thinning of the compact myocardium. *Mab21l2*-deficient embryonic hearts had decreased expression of genes that regulate cell proliferation and apoptosis of cardiomyocytes. These results show that *Mab21l2* functions during heart development by regulating the expression of such genes. *Mab21l2* was also expressed in the septum transversum mesenchyme (STM). Epicardial progenitor cells are localized to the anterior surface of the STM (proepicardium), and proepicardial cells migrate onto the surface of the heart and form the epicardium, which plays an important role in heart development. The rest of the STM is essential for the growth and survival of hepatoblasts, which are bipotential progenitors for hepatocytes and cholangiocytes. Proepicardial cells in *Mab21l2*-deficient embryos had defects in cell proliferation, which led to a small proepicardium, in which *α4 integrin* expression, which is essential for the migration of proepicardial cells, was down-regulated, suggesting that defects occurred in its migration. In *Mab21l2*-deficient embryos, epicardial formation was defective, suggesting that *Mab21l2* plays important roles in epicardial formation through the regulation of the cell proliferation of proepicardial cells and the migratory process of proepicardial cells. *Mab21l2*-deficient embryos also exhibited hypoplasia of the STM surrounding hepatoblasts and decreased hepatoblast proliferation with a resultant loss of defective morphogenesis of the liver. These findings demonstrate that *Mab21l2* plays a crucial role in both heart and liver development through STM formation.

## Introduction

Two distinct heart fields, the FHF (first heart field) and the SHF (second heart field), are derived from anterior lateral plate mesoderm and splanchnic mesoderm, respectively. In the mouse embryo, the FHF forms a crescent shape at E7.5, and the cells coalesce along the ventral midline to form an early heart tube at E8.0. At this stage, the SHF begins to migrate to the anterior and posterior ends of the heart tube to form the right ventricle, conotruncus and part of the atria. The heart tube then undergoes a process involving rightward looping and expansion of the myocardium, leading to the formation of the four-chambered heart [1], [2]. Ventricular myocardial differentiation and maturation are characterized by the formation of both the compact myocardium and the trabecular myocardium. The compact myocardium forms in the outer zone of the ventricular wall and trabecular myocardium forms as protrusions along the inside of the compact myocardium [3]. Proper development of the compact and the trabecular myocardium is required for normal contractility and circulation of blood, and the importance of the appropriate development of these structures is demonstrated by the fact that deletion of genes essential for the normal development of these structures causes embryonic lethality at approximately midgestation.

Mammalian liver development begins with hepatic induction from the ventral endoderm through signaling molecules such as FGFs and BMPs, derived from the cardiac mesoderm and septum transversum mesenchyme (STM), respectively. After specification of the hepatic endoderm, the tissue starts extending towards the midgut. Following formation of the liver bud, the basement membrane surrounding it is progressively disrupted, and hepatoblasts (bipotential progenitors for hepatocytes and cholangiocytes) delaminate from the bud and invade the surrounding STM as cords. At this stage, the STM functions as a source of signals for hepatoblast growth and survival, and inactivation of genes encoding those signaling molecules causes liver hypoplasia (reviewed in [4], [5]).

The STM is required not only for liver development but also for heart development in forming the proepicardium. The proepicardium is a region of the STM caudal to the heart, and cells in this mesenchymal structure spread over the surface of the myocardium to form the epicardium, the epithelial outer layer of the heart [6], [7]. Epicardial cells undergo an epithelial-to-mesenchymal transition, penetrate into the neighboring myocardium and differentiate into the cells required for cardiovascular development, such as myofibroblast, pericytes, and smooth muscle cell [8], [9], [10], [11]. Epicardial cells are important not only as a source of cells contributing to heart development, but also as a source of signaling molecules regulating proliferation of the myocardium. Signaling molecules including erythropoietin, retinoic acid and FGF are produced in the epicardium, and communication between the epicardium and myocardium through these molecules is required for normal myocardial proliferation [12], [13]. Therefore, the STM is an important tissue involved in both heart and liver development.

The *mab-21* gene was originally identified as a cell fate determinant in *Caenorhabditis elegans* and several studies have shown that *mab-21* is highly conserved across animal species, from vertebrates to invertebrates [14], [15], [16]. Two *mab-21* orthologues, *Mab21l1* and *Mab21l2*, have been found in species such as zebrafish, Xenopus, chicken, mouse, and human [14], [16], [17], [18], [19], [20]. MAB-21 family proteins in vertebrates have more than 90% amino acid sequence similarity, but do not have any known functional motifs. Thus predicting the molecular functions of MAB-21 proteins from their amino acid sequence is difficult. Several studies in vertebrates have shown that *Mab21l1* and *Mab21l2* expression overlap in the eye, midbrain, branchial arches, and limb buds during development. In mouse, *Mab21l1* is uniquely expressed in the lens and genital tubercle while *Mab21l2* expression is found in the retina, body wall and umbilical cord [14], [16], [19], [20], [21], [22].

We have previously shown that *Mab21l1*-deficient mice have defects in eye and preputial gland development, and that MAB21L1 functions cell-autonomously in the lens. Additionally, *Mab21l2*-deficient mice, which are embryonic lethal between E11.5 and E14.5 (lethality rate of homozygous mutant embryos: E11.5, 14%; E12.5, 65%; E13.5, 64%), have defects in eye and body wall formation. Thus, *Mab21l1* and *Mab21l2* function during eye development; *Mab21l1* is essential for lens placode development and *Mab21l2* is essential for retina development. *Mab21l1* and *Mab21l2* are thought to function downstream of *Pax6* in eye development [21], [23]. *Mab21l1* is also essential for the appropriate development of the preputial glands, while *Mab21l2* plays a crucial role in ventral body wall formation [21], [22].

We analyzed in detail, the expression of *Mab21l2* and embryonic tissues of *Mab21l2*-deficient mice. *Mab21l2* begins to be expressed in the heart region at approximately E8.5, and is expressed in both the trabecular and the compact myocardium at E9.5–E11.5, and deletion of *Mab21l2* results in a reduction in the amount of trabecular myocardium and a thinning of the compact myocardium. BrdU and TUNEL assays demonstrated that cell proliferation decreased and apoptosis increased in *Mab21l2* mutants. The *Mab21l2* transcript is also found in the STM from the region of the proepicardium to the region near the hepatoblasts at E9.5, and *Mab21l2* deletion causes defective morphogenesis of that tissue and results in defects in the epicardium and liver, demonstrating that *Mab21l2* is essential for heart and liver development through the STM. Taken together, these results indicate that *Mab21l2* is expressed in the cardiac field and the STM and plays essential roles during heart and liver development.

## Results

### *Mab21l2* is expressed in the trabecular and compact myocardium and is required for normal development of these tissues

**Figure 1:**
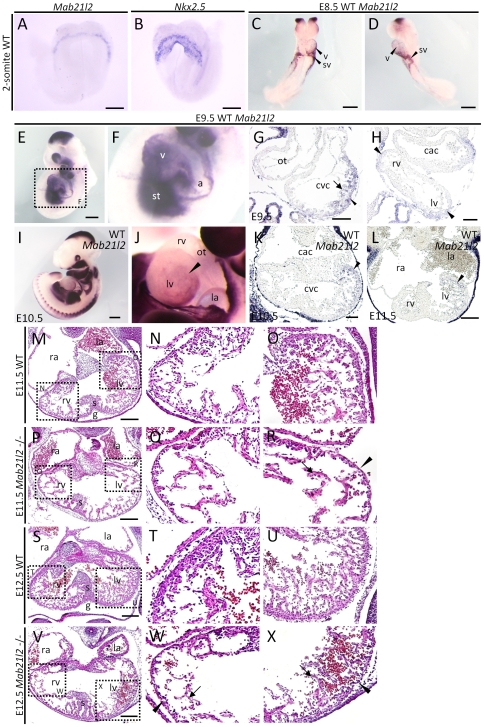
*Mab21l2* is required for the formation of the trabecular and compact myocardium. (A–F) Whole-mount *in situ* hybridization of 2-somite, E8.5 and E9.5 wild-type (WT) embryos for the indicated transcripts. (A, B) At the 2-somite stage, *Mab21l2* appeared to be expressed adjacent to the first heart field expressing *Nkx2.5*. (C and D) Expression of *Mab21l2* was detected in the ventricle, and sinus venosus at E8.5. (E and F) *Mab21l2* was expressed in the ventricle (v) and the septum transversum mesenchyme (st) at E9.5. Scale bar represents 60 µm(A, B) and 300 µm (C–E). (G and H) *In situ* hybridization analysis of *Mab21l2* in transverse paraffin sections of E9.5 WT embryonic heart. (G) *Mab21l2* is expressed in the trabecular myocardium (arrow) and the compact myocardium (arrowhead). (H) *Mab21l2* is expressed in the right and left ventricle (black arrowheads). Scale bars represent 50 µm (G) and 30 µm (H). (I and J) Whole-mount *in situ* hybridization of E10.5 WT embryos for *Mab21l2*. (J) Lateral view of the heart shown in (I). Scale bar in I represents 500 µm. (K and L) *In situ* hybridization analysis for *Mab21l2* in transverse paraffin sections of E10.5 (K) and E11.5 (L) WT embryonic hearts. (K and L) Expression of *Mab21l2* is seen on the left side of the left ventricle (arrowheads). Scale bars represent 50 µm in (K) and 100 µm (L). (M–X) Hematoxylin and eosin (H&E)-stained transverse sections of WT and *Mab21l2*-mutant embryonic hearts at E11.5 and E12.5. At E11.5, *Mab21l2* mutant embryos show defects in the left ventricle (R), but not in the right ventricle (Q), including reduced trabecular myocardium (arrow) and thin compact myocardium (arrowheads) compared with WT embryos (N and O). Defects were observed in the right (W) and left (X) ventricle in *Mab21l2*-mutants compared with WT embryos at E12.5 (T and U) (compact myocardium, arrowheads; trabecular myocardium, arrows). a, atrium; cac, common atrium chamber; cvc, common ventricular chamber; ot, outflow tract; g, ventricular groove; la, left atrium; lv, left ventricle; ra, right atrium; rv, right ventricle; v, ventricle; s, interventricular septum; st, septum transversum mesenchyme; sv, sinus venosus. Scale bar represents 100 µm (M, P, S and V).

Embryonic lethality in *Mab21l2*-deficient embryos first occurred at approximately E11.5. Living embryos were observed until E13.5 [22], but in E13.5 *Mab21l2* mutant embryos, the heartbeat was very weak. A known defect which results in embryonic lethality at around E11.5 is defective morphogenesis of the heart and cardiovascular system. Therefore, we examined whether the expression of *Mab21l2* was detected in those tissues before E11.5 in wild-type embryos by *in situ* hybridization. At the 2-somite stage, *Mab21l2* appeared to be expressed adjacent to the first heart field (identified by *Nkx2.5* expression, the first heart field marker), i.e. the presumptive STM [24] (Figure 1A, B). At E8.5, the expression of *Mab21l2* was detected in the ventricle and sinus venosus myocardium (Figure 1C, D). At E9.5, *Mab21l2* was expressed in the STM (Figure 1E, F) and the ventricular myocardium, in both the trabecular and the compact myocardium (Figure 1G, S1A, B; *Nkx2.5*, myocardial marker). Especially, the expression was detected in the future right ventricle and left ventricle (Figure 1H black arrowheads). At E10.5 and E11.5, the expression of *Mab21l2* was largely detected in the myocardium of the left ventricle, especially in the left side of the structures. The expression was strongest in the region between the atrium and ventricle (Figure 1I–L). At E12.5, the expression of *Mab21l2* was only detectable at low levels in the trabecular region of the left ventricle (Figure S2A, B). The expression of *Mab21l1*, another *mab-21* orthologue, was not detected in the heart region.

To study the contribution of *Mab21l2* expression to heart development, we examined hematoxylin and eosin (H&E) stained sections of *Mab21l2*-deficient embryos, and found defects in the heart region. In some *Mab21l2* embryos (3/32 embryos) at E10.5, the compact myocardium of the left ventricle was thinner (Figure S3H, J) than in wild-type embryos (Figure S3G, I) and at E11.5, in addition to the thin layer of compact myocardium, *Mab21l2*-deficient embryos had a reduced trabecular myocardium in the left ventricle (Figure 1R) compared to wild-type embryos (Figure 1O), although defects were not grossly observed in the right ventricle of *Mab21l2*-deficient embryos (Figure 1Q) when they were compared to wild-type embryos (Figure 1N) (After E11.5, defects in the heart region were observed in all *Mab21l2*-deficient embryos.). At E12.5, defects in the compact and trabecular myocardium were observed not only in the left, but also in the right ventricle of *Mab21l2*-deficient embryos (Figure 1W, X). Defective morphogenesis of the atrium was not observed in *Mab21l2*-deficient embryos at E10.5–E12.5 (Figure S3H, 1P, V). No defects in the heart region, including the endocardium and myocardium, were observed at E8.5 (Figure S3A, B) or E9.5 (Figure S3C–F). These results demonstrate that *Mab21l2* has an important role in the formation of the trabecular and compact myocardium by E11.5.

### BrdU and TUNEL assays demonstrate that the deletion of *Mab21l2* causes decreased cell proliferation and increased apoptosis

**Figure 2:**
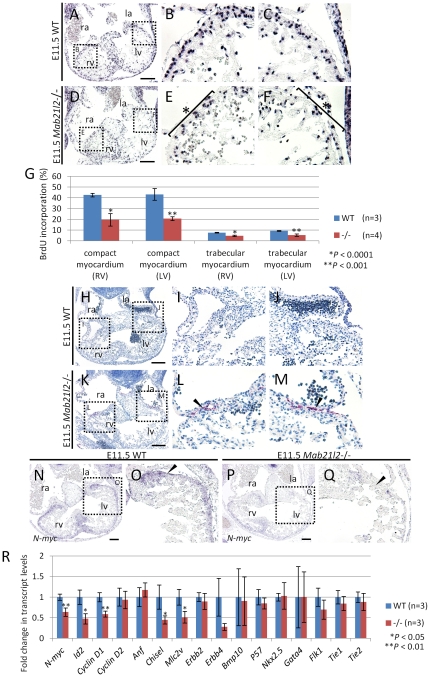
Decreased proliferation and increased apoptosis in *Mab21l2* mutant embryos. (A–F) BrdU assay on transverse paraffin sections of E11.5 WT and *Mab21l2* mutant (−/−) embryos. BrdU staining shows decreased cell proliferation in the trabecular myocardium and compact myocardium (asterisk) of left (F) and right ventricle (E) in *Mab21l2* mutant (D–F) compared to WT embryos (A–C). (G) Quantification of BrdU incorporation. The percentage of BrdU-positive myocardial cells was calculated by dividing the number of BrdU-positive myocardial cells by that of total myocardial cells identified by hematoxylin staining. The values show means of the proportions of BrdU-positive nuclei. Error bars represent the standard deviation and p values were calculated using the two-tailed Student's t test. (H–M) TUNEL assay in transverse paraffin sections of E11.5 embryos. Increased apoptosis is seen in *Mab21l2* mutant embryonic hearts (K–M) compared to WT (H–J). Arrowheads indicate TUNEL-positive cells (red). (N–Q) *In situ* hybridizations on transverse paraffin sections of E11.5 WT and *Mab21l2* mutant embryonic hearts for *N-myc*. *N-myc* expression was reduced in the compact myocardium of *Mab21l2* mutants (P and Q) compared to WT embryos (N and O), especially in the dorsal region of the left ventricle (arrowheads). la, left atrium; lv, left ventricle; ra, right atrium; rv, right ventricle. Scale bars represent 100 µm (A, D, H, K) and 50 µm (N and P). (R) Quantitative RT-PCR for genes regulating cell proliferation and apoptosis (*N-myc*, *Id2*, *Cyclin D1*, *Cyclin D2*), marker genes of myocardial differentiation (*Anf*, *Chisel*, *Mlc2v*), genes essential for the development of the trabecular myocardium (*Erbb2*, *Erbb4*, *Bmp10*), genes essential for heart development (*Nkx2.5*, *Gata4*), and endocardial transcripts (*Flk1*, *Tie1*, *Tie2*) normalized to *Gapdh* expression using RNA from dissected hearts of E11.5 WT and *Mab21l2* mutant (−/−) embryos. Error bars represent standard deviation of the mean (WT, n = 3; *Mab21l2* (−/−), n = 3). P values were calculated using the unpaired two-tailed Student's t test.

The reduced trabecular myocardium and thin compact myocardium suggest that *Mab21l2*-deficient embryos have defects in the regulation of cell proliferation and/or apoptosis in these regions after E10.5 or E11.5. The proportions of proliferating cells and apoptotic cells in the heart region were examined using 5-bromo-2′-deoxy-uridine (BrdU) and terminal deoxynucleotidyl transferase-mediated dUTP nick-end labeling (TUNEL) assays, respectively. At E10.5, BrdU incorporation was unchanged between wild-type and *Mab21l2* mutant hearts, suggesting that the proliferation of myocardial cells was normal (Figure S4). At E11.5, the BrdU assay showed that the number of cells incorporating BrdU in the trabecular and compact myocardium was reduced in *Mab21l2* mutant embryos (compact myocardium of right ventricle, <20%, and left ventricle, <20%; WT n = 3, *Mab21l2* −/− n = 4; trabecular myocardium of right ventricle, <5%, and left ventricle, <6%; Figure 2D–F) compared to wild-type embryos (Figure 2A–C). BrdU-positive cells were reduced in the ventricular myocardium lying between the atrium and ventricle, indicating that *Mab21l2*-deficient embryos have decreased cell proliferation in that region. At E10.5, TUNEL-positive cells were not generally detected in the heart region of *Mab21l2* mutant embryos (Figure S5D–F). However, in some embryos (1/8 embryos), TUNEL-positive cells were detected in the outermost layer of the myocardium in *Mab21l2* mutant embryos (Figure S5G–I). At E11.5, the TUNEL assay showed that TUNEL-positive cells were not detected in the wild-type embryos (Figure 2H–J), but were detected in the outermost layer of the myocardium in *Mab21l2*-deficient embryos (Figure 2K–M), suggesting that the deletion of *Mab21l2* results in apoptotic cell death in that region. These results demonstrate that decreased cell proliferation and increased apoptosis generally occur at around E11.5 and that defective morphogenesis in the trabecular and compact myocardium results from dysregulation of cell proliferation and apoptosis.

### The expression of *Mab21l2* is required for the transcription of the gene regulating cell proliferation and apoptosis

Defects in cell proliferation and apoptosis occurred at around E11.5. To understand the molecular basis for these defects, we examined the expression of genes known to regulate these processes using E11.5 embryo samples. *N-myc* (*Mycn* - Mouse Genome Informatics), which is involved in the regulation of cell proliferation and apoptosis [25], is expressed in the compact myocardium between E10.5 and E12.5, and the reduced expression of *N-myc* results in the thinning of the compact myocardium [26], [27], [28], [29], [30]. *N-myc* expression in the compact myocardium in wild-type and *Mab21l2*-deficient embryos was examined at E11.5 using *in situ* hybridization and quantitative RT-PCR. *N-myc* expression in that region was reduced, especially in the region where cell proliferation was decreased and apoptosis was increased (37%±10% lower) (Figure 2P, Q, R). Decreased expression of *Cyclin D1* (*Ccnd1* - Mouse Genome Informatics) [31] and *Id2* [32], [33], both *N-myc* target genes, was also observed in the heart region (*Id2*, 53%±12% lower; *Cyclin D1*, 42%±7% lower) (Figure 2R), suggesting that *Mab21l2* may regulate cell proliferation and apoptosis in the compact myocardium through *N-myc* expression and via a subset of *N-myc* target genes, such as *Cyclin D1* and *Id2*.

*Nkx2.5* is required for terminal differentiation of cardiomyocytes, including the establishment and/or maintenance of a gene expression program in the ventricles [34], [35], [36]. *Gata4* is required for heart tube formation, although *Gata4* expression is not essential for specifying the cardiac cell lineages [34], [35], [36]. These genes were normally expressed in *Mab21l2* mutant embryos (Figure 2R), suggesting that the deletion of *Mab21l2* does not affect cardiac specification and heart tube formation. In *Mab21l2*-mutant hearts, the expression of differentiation marker genes was decreased, such as the molecular marker for chamber myocardium, *Chisel* (*Smpx* - Mouse Genome Informatics) [37], and an early myocardial differentiation marker, *Mlc2v* (*Myl2* - Mouse Genome Informatics) (*Chisel*, 56%±9% lower; *Mlc2v*, 49%±13% lower) (*Chisel*, Figure 2R, Figure S6C, D; *Mlc2v*, Figure 2R, Figure S6G, H). However, if defects in myocardial differentiation occur, defects in heart development would also occur at much earlier stages. Therefore, decreased expression of myocardial differentiation markers is a secondary effect of decreased cell proliferation. *Erbb2* and *Erbb4* are expressed in the myocardium, and the signaling pathway activated through the ligand NRG1 and the ERBB2/ERBB4 receptor is essential for the development of the trabecular myocardium [38], [39], [40]. The expression of *Erbb2* was unchanged in *Mab21l2*-deficient embryos (Figure 2R), but that of *Erbb4* was reduced in the ventricle of *Mab21l2*-deficient embryos (73%±8% lower levels; Figure 2R, Figure S6K, L), although this difference could not be statistically confirmed. *Bmp10* is expressed after E8.75, and, by E11.5, the expression of *Bmp10* is detected primarily in the trabecular region. *Bmp10* is required for promoting proliferative activity in myocardial cells through transcriptional regulation of *P57* (*Cdkn1c* - Mouse Genome Informatics) [41], which is an important negative regulator involved in cell cycle exit [42], as well as maintenance of the expression of several important cardiogenic factors, such as *Nkx2.5* and *Mef2c* [41]. However, the expression of *Bmp10* and *P57* was not altered in *Mab21l2* mutant embryos at E11.5 (Figure 2R). The endocardial expression of *Flk1* (*Kdr* - Mouse Genome Informatics), *Tie1*, and *Tie2* (*Tek* - Mouse Genome Informatics) is required for trabeculation [43], [44], [45], [46]. The expression of these genes was unchanged in *Mab21l2* mutants (Figure 2R), suggesting that defective morphogenesis of the trabecular myocardium does not result from defects in endocardial development. These results suggest that reduced expression of *Erbb4* has an effect on trabecular development.

### *Mab21l2* is expressed in the proepicardium and plays a crucial role in the formation of this region and of the epicardium

**Figure 3:**
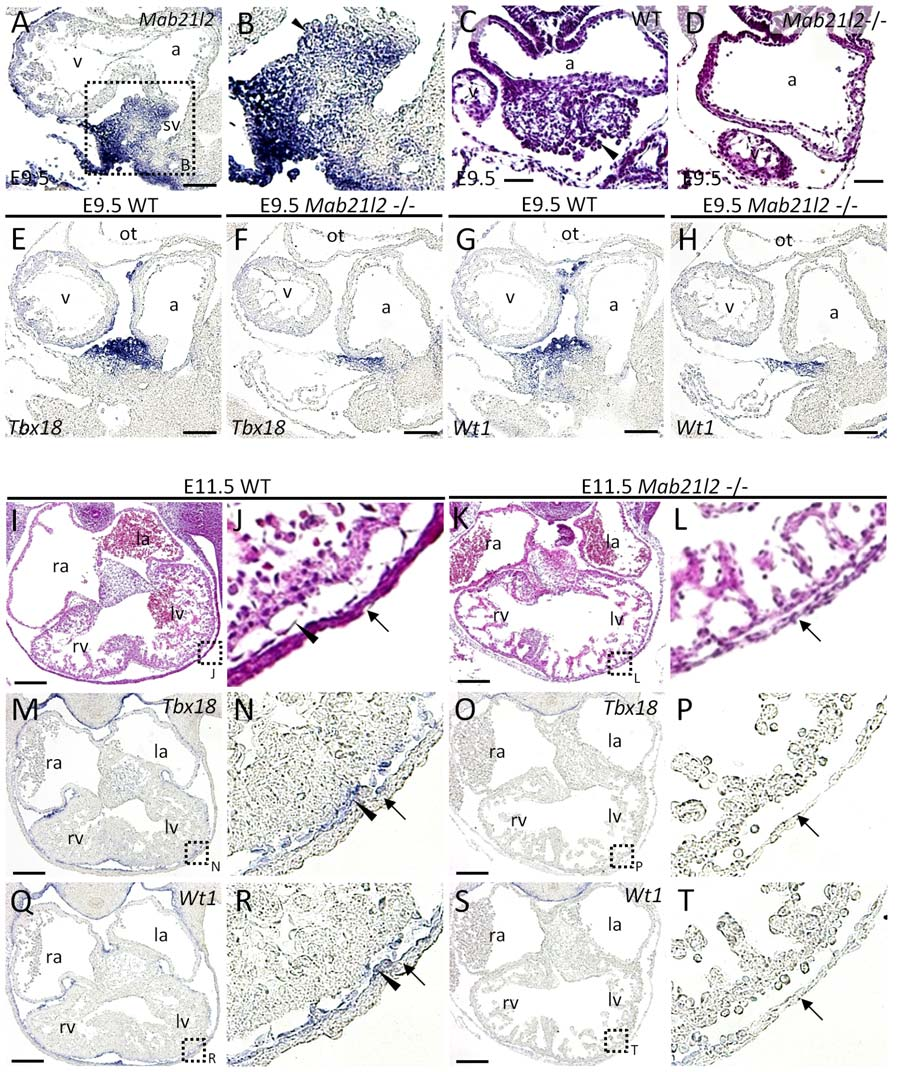
*Mab21l2* is required for the formation of the proepicardium and the epicardium. (A,B, E–H, M–T) *In situ* hybridizations of sagital (A, B, E–H) and transverse (M–T) paraffin sections of E9.5 and E11.5 WT and *Mab21l2* mutant embryos for the transcripts indicated. (C, D, I–L) H&E-stained transverse sections of E9.5 and E11.5 WT and *Mab21l2* mutant embryos. *Mab21l2* expression was detected in the proepicardium (arrowhead) of E9.5 WT embryos (A and B). Compared to WT embryos (C), the proepicardium (arrowhead) was absent in *Mab21l2* mutant embryos (D). Expression of the proepicardial and epicardial marker genes, *Tbx18*, and *Wt1*, was compared between WT ([E] *Tbx18*; [G] *Wt1*), and *Mab21l2* mutant embryos ([F] *Tbx18*; [H] *Wt1*), showing that the proepicardium is significantly small in *Mab21l2* mutant embryos. In E11.5 WT embryos (I and J), the epicardium (arrowhead) occurs between the compact myocardium and the body wall (arrows), but in *Mab21l2* mutant embryos (K and L), the epicardium is absent. The expression of epicardial marker genes was detected in E11.5 WT epicardium ([M and N] *Tbx18*; [Q and R] *Wt1*), arrowheads), but not in *Mab21l2* mutant embryos ([O and P] *Tbx18*; [S and T] *Wt1*), between the compact myocardium and the body wall (arrows). a, atrium; v, ventricle; la, left atrium; lv, left ventricle; ra, right atrium; rv, right ventricle; sv, sinus venosus. Scale bars represent 30 µm (C and D), 50 µm (A, E–H), and 100 µm (I, K, M, O, Q and S).

Next, it was examined whether defects occurred in the formation of the tissue adjacent to the ventricular myocardium: the endocardium, which lines the inside of the heart, and the epicardium, which covers the outside. The endocardium is formed in precardiac mesoderm [47], [48], [49], and signaling from the endocardium to the ventricular myocardium is required to initiate the conversion of the myocardial layer of the primitive heart tube into a thickened myocardial wall capable of contraction. The epicardium is formed after E10.5 and is required for optimal myocardial cell proliferation, leading to the formation of the normally contracting myocardium. *Mab21l2* expression was not detected in the endocardium or epicardium between E9.5–E12.5 (Figure S1A, B, S2A, B). *Mab21l2* was expressed in the proepicardium at E9.5 (Figure 1E, F, 3A, B). To study the effect of *Mab21l2* expression on proepicardium formation, we examined whether the proepicardium was normally formed in *Mab21l2* mutant embryos. H&E staining showed defective morphogenesis of the proepicardium (Figure 3D). Next, the expression of proepicardial and epicardial marker genes, such as *Wt1* [50], and *Tbx18* [51] in *Mab21l2*-deficient embryos was examined. The proepicardium (*Tbx18* ^+^, *Wt1* ^+^ region) was significantly small in *Mab21l2* mutants (*Tbx18*, Figure 3F; *Wt1*, Figure 3H), demonstrating that defects in proepicardium formation occurred in *Mab21l2* mutant embryos. These results suggest that *Mab21l2* plays a critical role in the formation of the proepicardium.

The defective morphogenesis of the proepicardium could result in defects in the epicardium. H&E staining of heart sections of *Mab21l2*-deficient embryos suggested that the epicardium was not formed (Figure 3K, L). To confirm the absence of the epicardium, the expression of *Tbx18* and *Wt1* was examined in *Mab21l2* mutant embryos. The expression was not detected around the heart (*Tbx18*, Figure 3O, P; *Wt1*, Figure 3S, T), demonstrating that the epicardium is not formed by E11.5. These results suggest that *Mab21l2* contributes to the formation of the epicardium through the formation of the proepicardium.

### Defective morphogenesis of the epicardium results from reduced expression of *α4 integrin* and decreased cell proliferation in the small proepicardium

**Figure 4:**
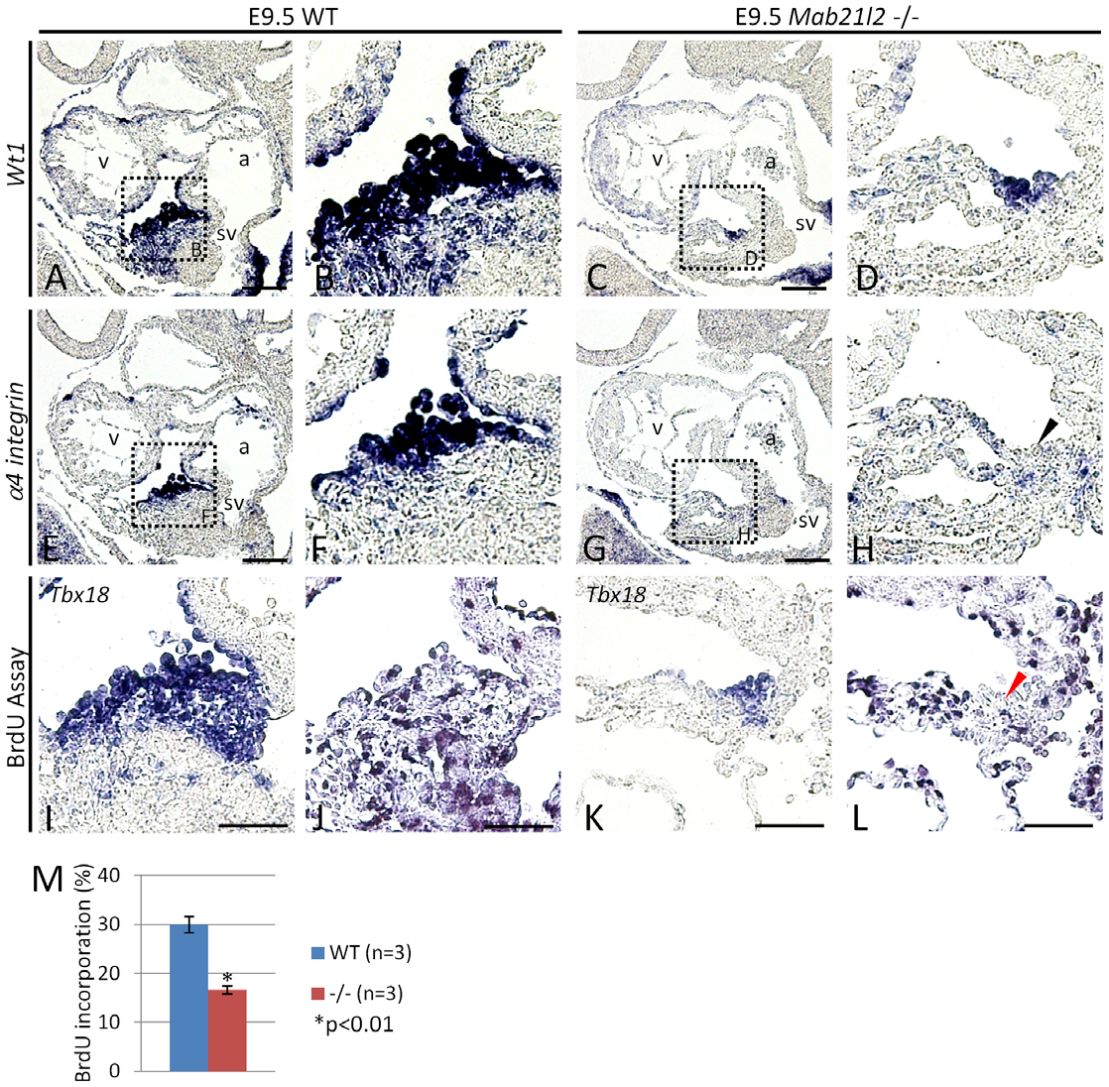
In the remaining proepicardium, *α4 integrin* expression and cell proliferation are reduced. (A–H, I and K) *In situ* hybridizations of sagital paraffin serial sections of E9.5 WT and *Mab21l2* mutant embryos for the transcripts indicated. In WT embryos, *α4 integrin* (F) was expressed in *Wt1*-positive proepicardial cells (B). In *Mab21l2*-mutant embryos, the expression of *α4 integrin* (H; black arrowhead) was significantly reduced in the *Wt1*-positive proepicardial cells (D). (J and L) BrdU assay on sagital paraffin serial sections of E9.5 from the WT and *Mab21l2* mutant (−/−) embryos. BrdU staining shows decreased cell proliferation in the *Tbx18*-positive proepicardial cells in the *Mab21l2* mutant (L; red arrowhead) compared to WT embryos (J). (M) Quantification of BrdU incorporation. The percentage of BrdU-positive proepicardial cells was calculated by dividing the number of BrdU-positive proepicardial cells by that of the total proepicardial cells identified by *Tbx18* expression. The values show means of the proportions of BrdU-positive nuclei. Error bars represent the standard deviation and p values were calculated using the two-tailed Student's t test. a, atrium; v, ventricle; sv, sinus venosus. Scale bars represent 50 µm (A, C, E, G) and 30 µm (I–L).

In E9.5 *Mab21l2* mutant embryos, the small proepicardium is present, but the epicardium is not formed by E11.5. Among mutants that have defects in epicardium formation, *α4 integrin* (*Itga4* - Mouse Genome Informatics) and *Vcam-1* (Vascular cell adhesion molecule-1) mutants have been previously reported [52], [53], [54]. *α4 integrin* encodes the α4 subunit of α4β1 integrin, a cell adhesion receptor, which binds to fibronectin [55], [56] and VCAM-1 [57], [58]. *α4 integrin* is expressed in the proepicardium and *Vcam-1* is expressed in the myocardium. In *α4 integrin* and *Vcam-1* mutant embryos, the proepicardium is normally formed by E9.5, and by E10.5, the epicardium is also normally formed in *α4 integrin* mutant embryos, however, by E11.5 the epicardium is absent from both mutant embryos [52], [54], demonstrating that the interaction through *α4 integrin* and *Vcam-1* is required for maintenance of cell adhesion of epicardial cells that have migrated to the surface of the heart. In another *α4 integrin* mutant line, epicardium formation did not occur by E10.5, suggesting that *α4 integrin* is also required for proepicardial cell migration from the proepicardium to the surface of the heart, and particularly for proepicardial cell adhesion to the myocardium when proepicardial cells reach this tissue [53]. In the remaining proepicardium and the myocardium, the expression of *α4 integrin* and *Vcam-1* was examined. The expression of *Vcam-1* in the myocardium was unchanged (Figure S7A, B), but that of *α4 integrin* was significantly reduced in the remaining proepicardium (*Wt1* ^+^ region) (*Wt1*, Figure 4C, D; *α4 integrin*, Figure 4G, H). To confirm that the epicardium was absent at E10.5, the expression of *Tbx18*, and *Wt1* was examined in *Mab21l2* mutant embryos. Expression was not detected around the heart (*Tbx18*, Figure S8C, D; *Wt1*, Figure S8G, H), suggesting that the epicardium is not formed at E10.5. These results suggest that reduced expression of *α4 integrin* in the remaining proepicardium results in defects of the migration of proepicardial cells, leading to defective morphogenesis of the epicardium by E10.5. Next, cell proliferation and apoptosis in the remaining proepicardium were examined using BrdU and TUNEL assays, respectively. The number of cells incorporating BrdU in the remaining proepicardium expressing *Tbx18* was lower in *Mab21l2* mutant embryos (<16%; WT n = 3, *Mab21l2* −/− n = 3; Figure 4L, M) than in wild-type embryos (Figure 4J, M). However, TUNEL-positive cells were not detected in the remaining proepicardium (data not shown). Taken together, the results suggest that defective morphogenesis of the proepicardium results from dysregulation of cell proliferation in proepicardial cells.

### *Mab21l2* is expressed in the septum transversum mesenchyme near hepatoblasts and plays an essential role in the formation of the liver

**Figure 5:**
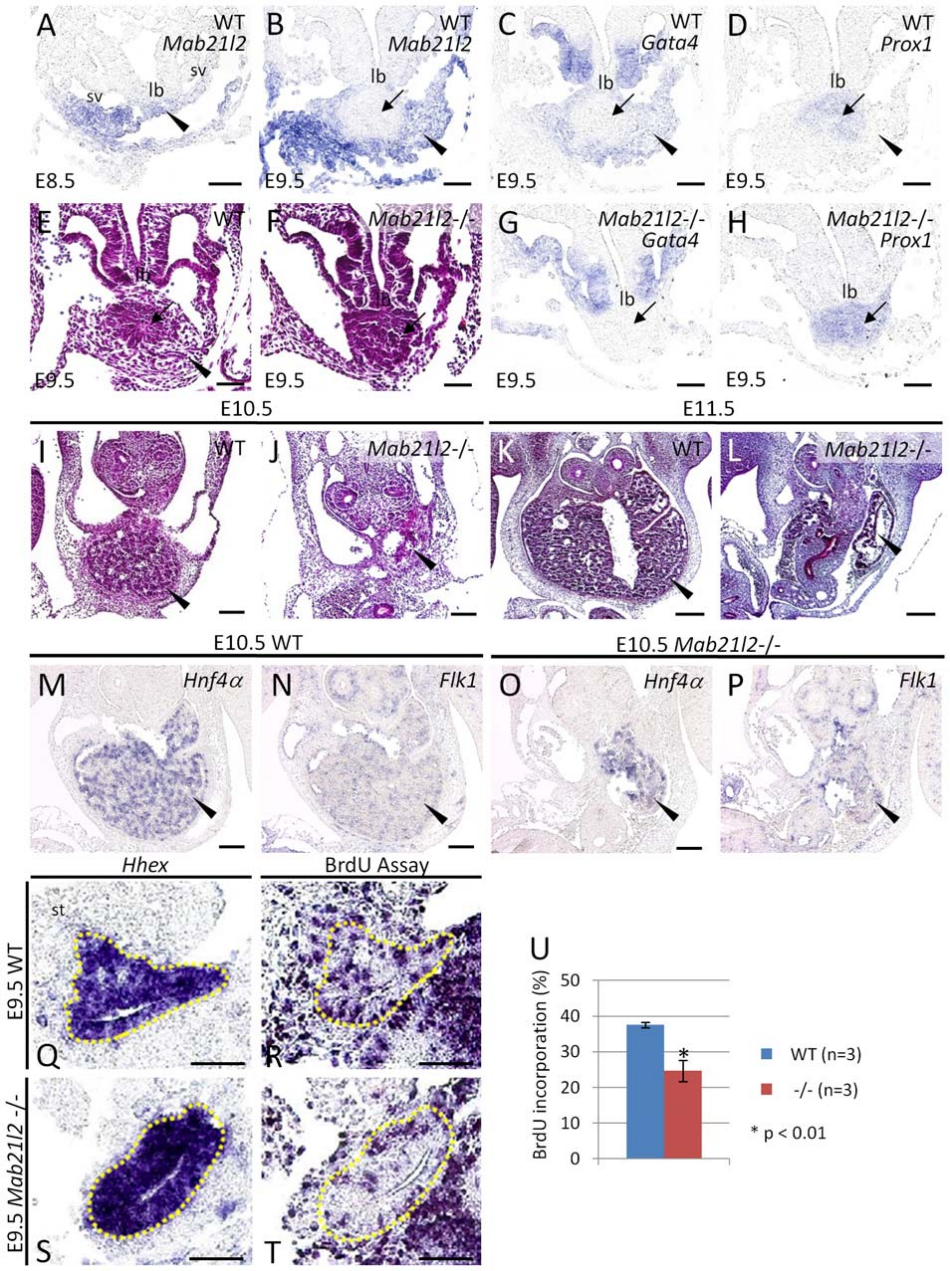
*Mab21l2* is required for the formation of the septum transversum mesenchyme near hepatoblasts and for normal liver development. (A–D, G, H, M–P) *In situ* hybridizations of transverse paraffin sections of E8.5, E9.5 and E10.5 WT and *Mab21l2* mutant embryos, for the transcripts indicated. (Q and S) *In situ* hybridizations of sagital paraffin sections of E9.5 WT and *Mab21l2* mutant embryos for *Hhex*. (E, F, and I–L) H&E-stained transverse sections of E9.5, E10.5, and E11.5 WT and *Mab21l2* mutant embryos. (R and T) BrdU assay on sagital paraffin serial sections of E9.5 from the WT and *Mab21l2* mutant (−/−) embryos. (U) Quantification of BrdU incorporation. The percentage of BrdU-positive hepatic cells was calculated by dividing the number of BrdU-positive hepatic cells by that of the total hepatic cells identified according to *Hhex* expression. The values show means of the proportions of BrdU-positive nuclei. Error bars represent the standard deviation and p values were calculated using the two-tailed Student's t test. *Mab21l2* expression was detected in the STM at E8.5 (A). Comparing the expression of *Mab21l2* (B), *Gata4*, an STM marker (C) and *Prox1*, a hepatoblast marker (D) in E9.5 WT embryos indicated that *Mab21l2* is expressed in the STM (arrowheads) near hepatoblasts (arrows) at E9.5. Defective morphogenesis was observed in the region near the liver bud in E9.5 *Mab21l2* mutant (F) compared to WT embryos (E). *Gata4* was not expressed in this region (G) but *Prox1* was expressed (H). *Mab21l2* mutant embryos at E10.5 (J) and E11.5 (L) show defective morphogenesis of the liver (arrowheads) compared to WT embryos (I and K). At E10.5, the expression of *Hnf4α* (hepatoblast marker) and *Flk1* (endothelial marker) was detected in *Mab21l2* mutant embryos (O and P). BrdU staining shows decreased cell proliferation in the *Hhex*-positive hepatic region in the *Mab21l2* mutant (T) compared to WT embryos (R). lb, liver bud; st, septum transversum mesenchyme; sv, sinus venosus. Scale bar represents 30 µm (A–H, Q–T), 50 µm (I, J, M–P) and 100 µm (K, L).

Next, we examined whether *Mab21l2* was expressed, not only in the proepicardium, at the anterior of the STM, but also in the rest of the STM. At E8.5, the expression of *Mab21l2* was detected in the STM and sinus venosus myocardium (Figure 5A). At E9.5, the STM surrounds the hepatoblasts; therefore, *Mab21l2* expression was examined in this region (STM marker: *Gata4*), and in the hepatoblasts surrounding the liver bud (hepatoblast marker: *Prox1*). *Mab21l2* expression (Figure 5B; Figure 3A, B) overlapped almost completely with *Gata4* expression (Figure 5C), but not with *Prox1* (Figure 5D), showing that *Mab21l2* is expressed in the caudal region of the STM, but not in hepatoblasts. The expression of *Mab21l1* was not expressed in the STM.

To study the effect of *Mab21l2* expression on the formation of the caudal region of the STM, H&E-stained sections of *Mab21l2*-deficient embryos were examined. In *Mab21l2* mutant embryos, emergence of the liver bud occurred normally, although defective morphogenesis was observed around the liver bud (Figure 5F); however, the region from which the defects originated was unclear. To test whether these defects occurred in the STM or in hepatoblasts, the expression of *Gata4* and *Prox1* was examined in *Mab21l2* mutant embryos. *Prox1* expression was detected in hepatoblasts (Figure 5H), but *Gata4* was not (Figure 5G). Besides, *Tbx18* ^+^, *Wt1* ^+^ cells (proepicardial cells) were detected in *Mab21l2* mutant embryos at E9.5, but *Tbx18* ^−^, *Wt1* ^+^ cells (STM cells except proepicardial cells) were not (*Tbx18*, Figure 3F; *Wt1*, Figure 3H). These results show that the STM was absent from the region around the hepatoblasts at E9.5. These results suggest that *Mab21l2* plays an essential role in the formation not only of the proepicardium (the STM caudal to the heart), but also of the rest of the STM.

The caudal region of the STM functions as a source of signals for hepatoblast growth and survival, and the inactivation of genes encoding these signaling molecules causes liver hypoplasia [5], and therefore, defects in the STM would be expected to cause dysmorphogenesis of the liver. H&E-stained sections of *Mab21l2* mutant embryos at E10.5 and E11.5 suggested that defective morphogenesis of the liver had occurred (Figure 5J, L). To verify whether the remaining tissue was hepatic tissue, the expression of *Hnf4α* (hepatoblast marker) and *Flk1* (endothelial cell marker) was examined. The expression of both genes was detected in *Mab21l2* mutant embryos (Figure 5O, P), showing that hepatoblasts, surrounded by endothelial cells, were present in the small liver of *Mab21l2* mutant embryos. Next, BrdU and TUNEL assays were performed to examine whether decreased cell proliferation and/or increased apoptosis, respectively, occurred in the hepatic region. The number of cells incorporating BrdU in the hepatic region expressing *Hhex* was lower in *Mab21l2* mutant embryos (<24%; WT n = 3, *Mab21l2* −/− n = 3; Figure 5T, U) than in wild-type embryos (<37%; Figure 5R, U). However, no TUNEL-positive cells were detected in the hepatic region (data not shown). Taken together, the results suggest that defective STM morphogenesis results in a decrease in the signals promoting hepatoblast proliferation, leading to a small liver. Next, the expression of the endothelial marker, *Flk1*, and the hepatoblast markers, *Alb*, *Hhex*, and *Hnf4a* was examined in wild-type and mutant embryos. The expression of *Flk1* was unchanged in *Mab21l2* mutant embryos (Figure S9E), suggesting that the loss of the basement membrane occurred as normal. The expression of *Alb*, *Hhex* and *Hnf4a* was also unchanged (Figure S9F, H) suggesting that the initial hepatic induction occurred as normal and that defective morphogenesis of the STM does not affect hepatoblast differentiation at E9.5.

## Discussion

This study demonstrated that *Mab21l2* is expressed in the septum transversum mesenchyme (STM) including the proepicardium, and plays an essential role in the formation of that tissue. The STM is derived from the lateral-plate mesoderm. At E7.5, the presumptive STM is located at the junction between the visceral yolk sac and the amnion. This positional relationship is maintained during neural fold formation and foregut invagination, and, as a result, at E9.0 the STM is located caudal to the heart. Currently, *Gata4* is known to be essential for the formation of the proepicardium [59], and the phenotype of *Gata4* mutant embryos in proepicardium formation is the same as *Mab21l2* mutant embryos. However, the precise molecular mechanisms underlying the formation of the proepicardium remain unknown.

From the anterior surface of the STM, the proepicardium, epicardial progenitor cells migrate to the heart to form the epicardium. In the remaining proepicardium of *Mab21l2* mutant embryos, cell proliferation was decreased, suggesting that *Mab21l2* contributes to proepicardium formation by promoting the proliferation of proepicardial cells. Although a small proepicardium was present in *Mab21l2* mutant embryos, not even transient epicardial formation was observed at E9.5–E10.5, suggesting that defects also occur in the migration of proepicardial cells from the proepicardium to the heart surface in *Mab21l2* mutant embryos. *Vcam-1* was normally expressed in the myocardium, but the expression of *α4 integrin* was significantly reduced in the remaining proepicardium (*Wt1* ^+^ region). *α4 integrin* is essential for the migration of proepicardial cells onto the heart surface, suggesting that, in *Mab21l2* mutant embryos, proepicardial cells that reach the heart surface cannot adhere to the myocardium due to reduced expression of *α4-integrin*. Thus, during epicardial development, *Mab21l2* plays important roles in both proepicardium formation and proepicardial cell migration. Epicardial cells play a crucial role during heart development by providing a source of cells contributing to heart formation, such as cardiac fibroblasts, pericytes and smooth muscle cells [8], [9], [10], [11]. Although beginning to invade the myocardium at E11.5 [60], epicardial cells provide signaling molecules promoting the proliferation of the myocardial cells [12], [13]. Cell proliferation was decreased in E11.5 *Mab21l2*-mutant embryonic hearts, suggesting that myocardial defects and loss of the epicardium affect cardiomyocyte proliferation. Mice mutant in *Wt1*, *α4integrin* or *Vcam-1* has defects in epicardium formation, resulting in defects in heart development similar to *Mab21l2* mutant embryos, further demonstrating the importance of the epicardium for normal heart development. Taken together, these results suggest that *Mab21l2* plays an essential role during heart development through the formation of the proepicardium and the epicardium.

Embryonic lethality in *Mab21l2* mutant embryos occurred after E11.5, and the expression of *Mab21l2* was detected especially in the left ventricle at E10.5 and E11.5. In E11.5 *Mab21l2* mutant embryos, defective morphogenesis was observed not only in the left ventricle, but also in the epicardium. Epicardial cells, surrounding the right and left ventricle, promote cell proliferation of surrounding cardiomyocytes, and it is possible that defects in the epicardium have an effect on cardiomyocyte cell proliferation. If loss of the epicardium has a major influence on defects in the myocardium, these defects would be expected to occur in the right and left ventricle. However, defects were observed only in the left ventricle, suggesting that defective morphogenesis in the left ventricle at E11.5 largely results from loss of function of *Mab21l2* in the myocardium. At E12.5, defects in the heart region were observed in the right and left ventricle. At E11.5 and E12.5, the expression of *Mab21l2* was not detected in the right ventricle, suggesting that defects in the heart region at E12.5 largely result from loss of the epicardium. The trabecular and compact myocardium are essential for maintaining adequate blood circulation, suggesting that embryonic lethality observed in *Mab21l2* mutants at approximately E11.5 may be caused by defective morphogenesis of these regions.

The STM is important not only as a source of progenitors of the epicardium, but also as a source of various signaling components. Prior to and during hepatic induction, inductive signals such as BMPs are expressed in the STM and initiate the liver gene program in the proximal endoderm in conjunction with FGFs from the cardiac mesoderm. In *Mab21l2* mutant embryos, marker genes of hepatic induction, such as *Alb*, *Hhex*, are normally expressed in the hepatic tissue at E9.5, suggesting that the induction of the liver gene program may normally occur, and at the stage when hepatic induction occurs, the STM may be present. After the induction of liver development, the STM near hepatoblasts, at approximately E9.5, plays an essential role in regulating the growth and survival of hepatoblasts (reviewed in [4], [5]). Reduced cell proliferation was observed in the hepatic region in *Mab21l2* mutant embryos at E9.5. *Flk1* expression in *Mab21l2* mutant embryos suggests that the endothelial basement membrane degrades as expected, and *Prox1*, which is required for the migration of hepatoblasts into the STM from the liver bud [61], was expressed normally in hepatoblasts in *Mab21l2* mutant embryos (Figure 5H), suggesting that hepatoblasts may be clustered in the liver bud in these embryos, because the STM into which hepatoblasts migrate was absent. Other hepatoblast markers, such as *Alb*, *Hhex* and *Hnf4a*, were expressed normally, suggesting that defective morphogenesis of the STM does not affect hepatoblast differentiation at E9.5. Thus, defective liver morphogenesis in *Mab21l2* mutant embryos was caused by loss of the signals from the STM that are required for hepatoblast growth. Taken together, these results show that *Mab21l2* plays an essential role in liver morphogenesis by mediating the formation of the STM. *Mab21l2* is expressed in the STM, not in surrounding hepatoblasts. Loss of function of *Mab21l2* results in defective morphogenesis of the STM, leading to defects in cell proliferation of hepatoblasts. These results suggest that *Mab21l2* has effects on liver development in a non-cell-autonomous manner.

The STM is a transient tissue that exists during embryogenesis from around E7.5 to E9.5, and is adjacent to the various organs or presumptive organ regions, such as the heart, liver and ventral pancreas. Similar to *Mab21l2*-deficient mice, *Gata4*-deficient mice lack the STM, including the proepicardium, at E9.5, but *Gata4* mutant mice die by approximately E10.0, and therefore the importance of the STM as a tissue during organogenesis after E10.5 has remained unknown [59], [62]. Fortunately, *Mab21l2* mutant mice survive after E10.5, allowing the contribution of the STM to organogenesis after E10.5 to be examined. In this study, we have shown that defects of the STM, including the proepicardium, resulted in the loss of the epicardium and defective morphogenesis of the heart and liver. These results demonstrate that the STM plays essential roles in the morphogenesis of the surrounding organs, such as the heart and liver, although the molecular functions of the MAB21L2 protein remain unclear. To uncover the molecular mechanism underlying STM morphogenesis, it will be necessary to analyze MAB21L2 protein function, which should help to provide a detailed understanding of how the STM functions during the morphogenesis of its surrounding organs.

## Materials and Methods

### Mice

The generation of mutant mice has been reported previously [22]. Mice were maintained in accordance with protocols approved by the Animal Care and Use Committee of the University of Tokyo.

### *In situ* hybridization

Whole-mount *in situ* hybridization was performed as described previously [63] at 65°C in 50% formamide containing 5× SSC. Paraffin sections were hybridized *in situ* at 65°C in 50% formamide, 20 mM Tris-HCl (pH 8.0), 300 mM NaCl, 0.2% Sarkosyl, 1× Denhart's solution, 10% dextran sulfate, and 0.5 mg/ml yeast tRNA. All probes were labeled with digoxigenin using standard procedures. Details for probes will be provided upon request.

### Histology

Embryos were dissected in phosphate-buffered saline (PBS) and fixed in 4% paraformaldehyde in PBS overnight at 4°C. The fixed embryos were dehydrated through a graded alcohol series and embedded in paraffin, sectioned (8 µm thick sections), and stained with hematoxylin and eosin.

### Detection of proliferating or apoptotic cells

Pregnant mice were injected intraperitoneally with 3 mg bromodexyuridine (BrdU). Embyros were sacrificed 2 h later and fixed in 4% paraformaldehyde in PBS overnight at 4°C. Sections (8 µm thick) of embryos embedded in paraffin were stained with an anti-BrdU antibody using the BrdU Labeling and Detection Kit II (Roche). TdT-mediated dUTP nick end labeling (TUNEL) analysis was performed as follows. Sections (8 µm thick) of embryos embedded in paraffin were incubated in 3% H_2_O_2_ for 15 min, ProK solution for 10 min, and then incubated in TdT reaction solution (0.2 mM fluorescein-12-dUTP (Roche), 0.2 mM dATP, 1 mM CoCl_2_, 30 mM Tris-HCl (pH 7.5), 140 mM sodium cacodylate, 40 U terminal deoxynucleotidyl transferase (Roche)). Fluorescein was detected with an alkaline phosphatase-conjugated anti-fluorescein antibody (Roche) in a solution containing a phosphatase substrate (Fast Red Tablets, Roche), and counter-stained with hematoxylin.

### Quantitative RT-PCR

Whole hearts were dissected from E11.5 embryos. The hearts were disrupted in Sepasol(R)-RNA II Super (Nacalai Tesque) and RNA was purified using standard methods. Synthesis of cDNAs was performed with 150 ng total RNA using M-MuLV Reverse Transcriptase (BioLabs). Quantitative real-time PCR was performed using the SYBR Premix Ex TaqTM (Takara Bio), on a Thermal Cycler Dice Real Time System TP800 (Takara Bio), according to the manufacturer's protocols. All results were normalized to the levels of glyceraldehyde-3-phosphate dehydrogenase (*Gapdh*) mRNA. The primers used in this study were as follows: *Anf*-Fw, TGTACAGTGCGGTGTCCAAC; *Anf*-Rv, CCTGCTTCCTCAGTCTGCTC; *Bmp10*-Fw, CTGAACTGCGGTTGTACACG; *Bmp10*-Rv, CTCCTCTCCTCCTCGCTACC; *Chisel*-Fw, CACCGGGAGTTCCTTCTATC; *Chisel*-Rv, TAGCCCTGCTCTCTGGATTG; *Cyclin D1*-Fw, GGGACATAGCATCACAGCAG; *Cyclin D1*-Rv, CCGGAGACTCAGAGCAAATC; *Cyclin D2*-Fw, ACCGCACACATAGGCTTCTC; *Cyclin D2*-Rv, ATAACACCTCCTGGGGCTTC; *Erbb2*-Fw, TCAGCCCCAGAGGATTACAG; *Erbb2*-Rv, CTGCTCCCAGGATATTCACC; *Erbb4*-Fw, AACAGCAGTACCGAGCCTTG; *Erbb4*-Rv, GGATAGACCGCAGGAAGGAG; *Flk1*-Fw, GGCCGAGTCTGTCTACCTTG; *Flk1*-Rv, CTTCCTTCCTCCCAGTCCAC; *Gata4*-Fw, TCTCACTATGGGCACAGCAG; *Gata4*-Rv, CAGACAGCACTGGATGGATG; *Id2*-Fw, CCCCAGAACAAGAAGGTGAC; *Id2*-Rv, ATGCAGGCTGACGATAGTGG; *Mlc2v*-Fw, TACCCACGGAGAAGAGAAGG; *Mlc2v*-Rv, CCAGAGCCAAGACTTCCTGT; *Nkx2.5*-Fw, TTGACGTAGCCTGGTGTCTC; *Nkx2.5*-Rv, CCCGGTCCTAGTGTGGAATC; *N-myc*-Fw, AATTGGTCCCCTGTCGAGTC; *N-myc*-Rv, CACCCAGCATCCCATAAGTC; *P57*-Fw, GGACGATGGAAGAACTCTGG; *P57*-Rv, AAAACCGTGGGCTGCTCTAC; *Tie1*-Fw, CTGCGATGACGAAGTGTACG; *Tie1*-Rv, CCCAACTGTAGTGCGATCTG; *Tie2*-Fw, ACCCACTGCCAAGAGATGTG; *Tie2*-Rv, AGATCCGCACGAGCTGTATG.

## Supporting Information

Figure S1 ***Mab21l2*** **is expressed in the myocardium.** (A–D) *In situ* hybridizations of *Mab21l2* (A and B) and *Nkx2.5* (C and D) in transverse serial paraffin sections of WT embryonic hearts at E9.5. Expression of *Mab21l2* was detected in the trabecular and compact myocardium expressing *Nkx2.5* (a marker for cardiomyocytes). en, endocardium; ot, outflow tract; v, ventricle; t, trabecular myocardium; c, compact myocardium. Scale bar represents 50 µm.
(TIF)

Figure S2 **The expression of** ***Mab21l2*** **at E12.5.** (A and B) *In situ* hybridizations of transverse paraffin sections of E12.5 WT embryonic hearts for *Mab21l2*. *Mab21l2* expression was detected at low levels in the trabecular myocardium (arrow), not in the epicardium (arrowhead) at E12.5 (A and B). la, left atrium; lv, left ventricle; ra, right atrium; rv, right ventricle. Scale bar represents 100 µm (A).
(TIF)

Figure S3 **Defective morphogenesis in the heart region is observed from E10.5.** (A–J) H&E-stained transverse sections of E8.5, E9.5 and E10.5 WT and *Mab21l2* mutant embryonic hearts. Defects in the heart region were not observed in *Mab21l2* mutant embryos (B, D and F) compared to WT embryos (A, C and E) at E8.5 (A and B) and E9.5 (C–F). However, thin compact myocardium was just visible in some *Mab21l2* mutant (H and J) compared to WT embryos (G and I) at E10.5 (G–J). cvc, common ventricular chamber; la, left atrium; lv, left ventricle; ot, outflow tract; ra, right atrium; rv, right ventricle. arrows, trabecular myocardium; arrowheads, compact myocardium. Scale bar represents 30 µm (A and B), and 50 µm (C, D, G and H).
(TIF)

Figure S4 **At E10.5, cell proliferation in the heart region of the** ***Mab21l2*** **-mutant is unchanged compared to that of WT embryos.** (A–F) BrdU assay on transverse paraffin sections of E10.5 WT and *Mab21l2* mutant (−/−) embryos. BrdU staining shows that cell proliferation in the heart region of the *Mab21l2*-mutant (D–F) is normal, compared to WT embryos (A–C). (G) Quantification of BrdU incorporation. The percentage of BrdU-positive myocardial cells was calculated by dividing the number of BrdU-positive myocardial cells by that of the total myocardial cells identified by hematoxylin staining. The values show means of the proportions of BrdU-positive nuclei. Error bars represent the standard deviation. la, left atrium; lv, left ventricle; ra, right atrium; rv, right ventricle. Scale bar represents 50 µm.
(TIF)

Figure S5 **At E10.5, apoptosis in the heart region is generally unchanged in** ***Mab21l2*** **-mutant compared to WT embryos.** (A–I) TUNEL assay on transverse paraffin sections of E10.5 embryos. (D–F) In *Mab21l2* –mutant embryos (D–F), apoptosis was not generally detected in the heart region, but in some *Mab21l2*-mutant embryos (G–I), increased apoptosis was observed. Arrowhead indicates TUNEL-positive cells (red). lv, left ventricle; ra, right atrium; rv, right ventricle. Scale bar represents 50 µm.
(TIF)

Figure S6 ***Mab21l2*** **mutants show defective expression of some myocardial differentiation markers and of** ***Erbb4*** **essential for ventricular trabeculation.** (A–L) *In situ* hybridizations of transverse paraffin sections of E11.5 WT and *Mab21l2* mutant embryonic hearts for the transcripts indicated. The expression of *Chisel* and *Mlc2v* (cardiomyocyte differentiation markers), was reduced in the myocardium of *Mab21l2* mutants (*Chisel* [C and D]; *Mlc2v* [G and H]; arrowheads) compared to WT embryos (*Chisel* [A and B]; *Mlc2v* [E and F]), especially in the dorsal region of the left ventricle (arrowheads). The expression of *Erbb4* (essential for trabecular myocardial development in the heart ventricle) was also reduced in the *Mab21l2* mutant myocardium (K and L) compared to WT embryos (I and J). la, left atrium; lv, left ventricle; ra, right atrium; rv, right ventricle. Scale bar represents 50 µm.
(TIF)

Figure S7 ***Vcam-1*** **expression was unchanged in** ***Mab21l2*** **-mutant myocardium.** (A and B) *In situ* hybridization analysis of *Vcam-1* in sagital paraffin sections of E9.5 WT and *Mab21l2* mutant embryos. (B) *Vcam-1* was normally expressed in the *Mab21l2*-mutant embryo myocardium compared with WT embryos (A). a, atrium; v, ventricle; sv, sinus venosus. Scale bars represent 50 µm.
(TIF)

Figure S8 **Defective morphogenesis of the epicardium occurred by E10.5.** (A–H) *In situ* hybridizations of transverse paraffin sections of E10.5 WT and *Mab21l2* mutant embryos for the transcripts indicated. The expression of epicardial marker genes was detected in E10.5 WT epicardium ([A and B] *Tbx18*; [E and F] *Wt1*), arrowheads), but not in *Mab21l2* mutant embryos ([C and D] *Tbx18*; [G and H] *Wt1*) between the compact myocardium and the body wall (arrows). lv, left ventricle; ra, right atrium; rv, right ventricle. Scale bar represents 50 µm.
(TIF)

Figure S9 **Defective morphogenesis of the STM does not affect the expression of the endothelial marker,** ***Flk1*** **, or the hepatoblast markers,** ***Alb*** **,** ***Hhex and Hnf4a*** **.** (A–H) *In situ* hybridization of transverse paraffin sections of E9.5 WT and *Mab21l2* mutant embryos for the transcripts indicated. *Flk1* expression was not altered in *Mab21l2* mutant embryos (E), compared to WT embryos (A). The expression of the hepatoblast markers, *Alb*, *Hhex* and *Hnf4α* also remained unaltered in *Mab21l2* mutant embryos (F–H), compared to WT embryos (B–D). lb, liver bud; arrowheads, septum transversum mesenchyme. Scale bar represents 30 µm.
(TIF)
