# Supplementary figures and images for: Mab21l2 Is Essential for Embryonic Heart and Liver Development
Source: PLoS One. 2012 Mar 8;7(3):e32991. doi: 10.1371/journal.pone.0032991 (PMC3297618; doi:10.1371/journal.pone.0032991)

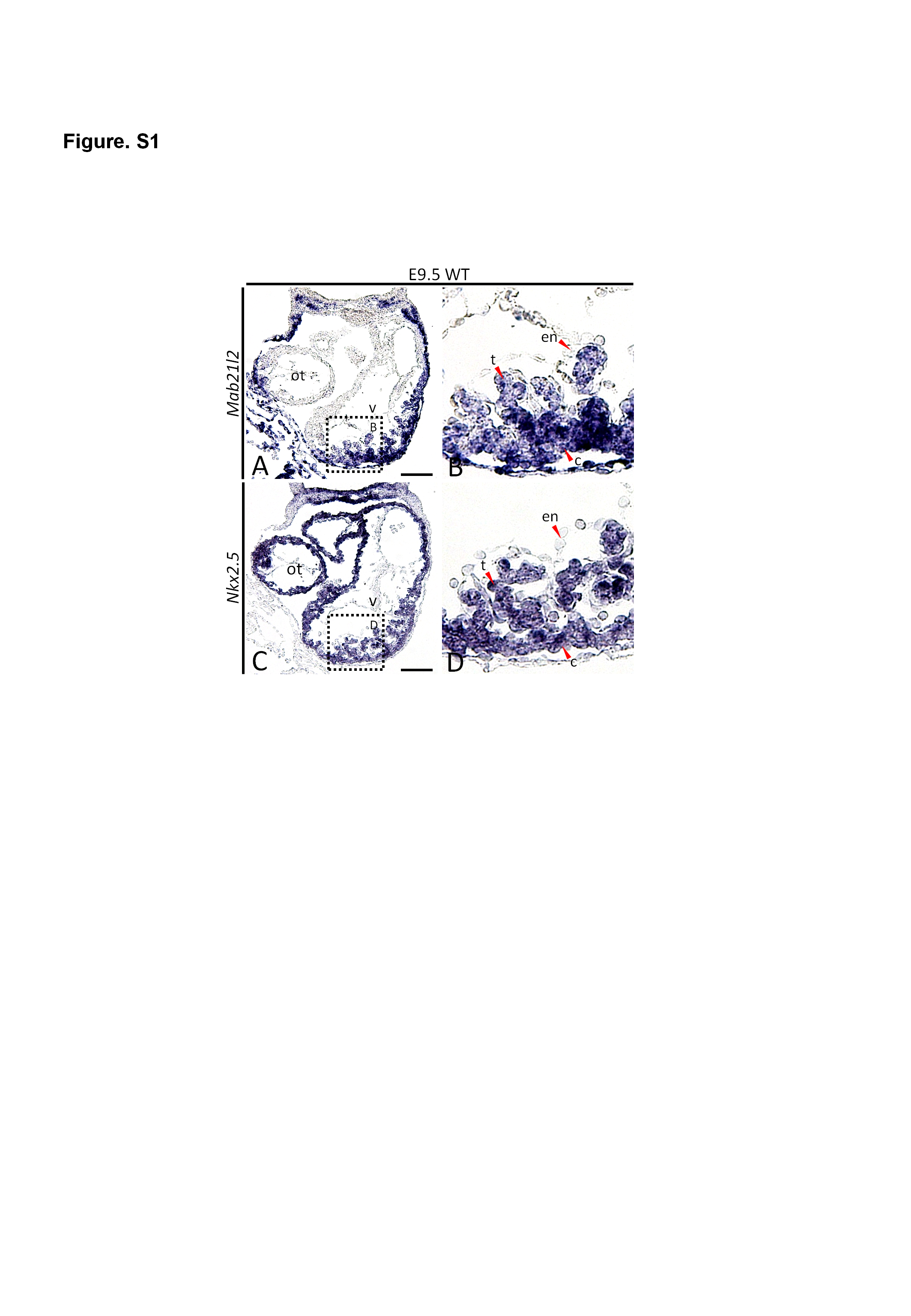

Supplement: Figure S1 — Mab21l2 is expressed in the myocardium. (A–D) In situ hybridizations of Mab21l2 (A and B) and Nkx2.5 (C and D) in transverse serial paraffin sections of WT embryonic hearts at E9.5. Expression of Mab21l2 was detected in the trabecular and compact myocardium expressing Nkx2.5 (a marker for cardiomyocytes). en, endocardium; ot, outflow tract; v, ventricle; t, trabecular myocardium; c, compact myocardium. Scale bar represents 50 µm. (TIF) [file pone.0032991.s001.tif]

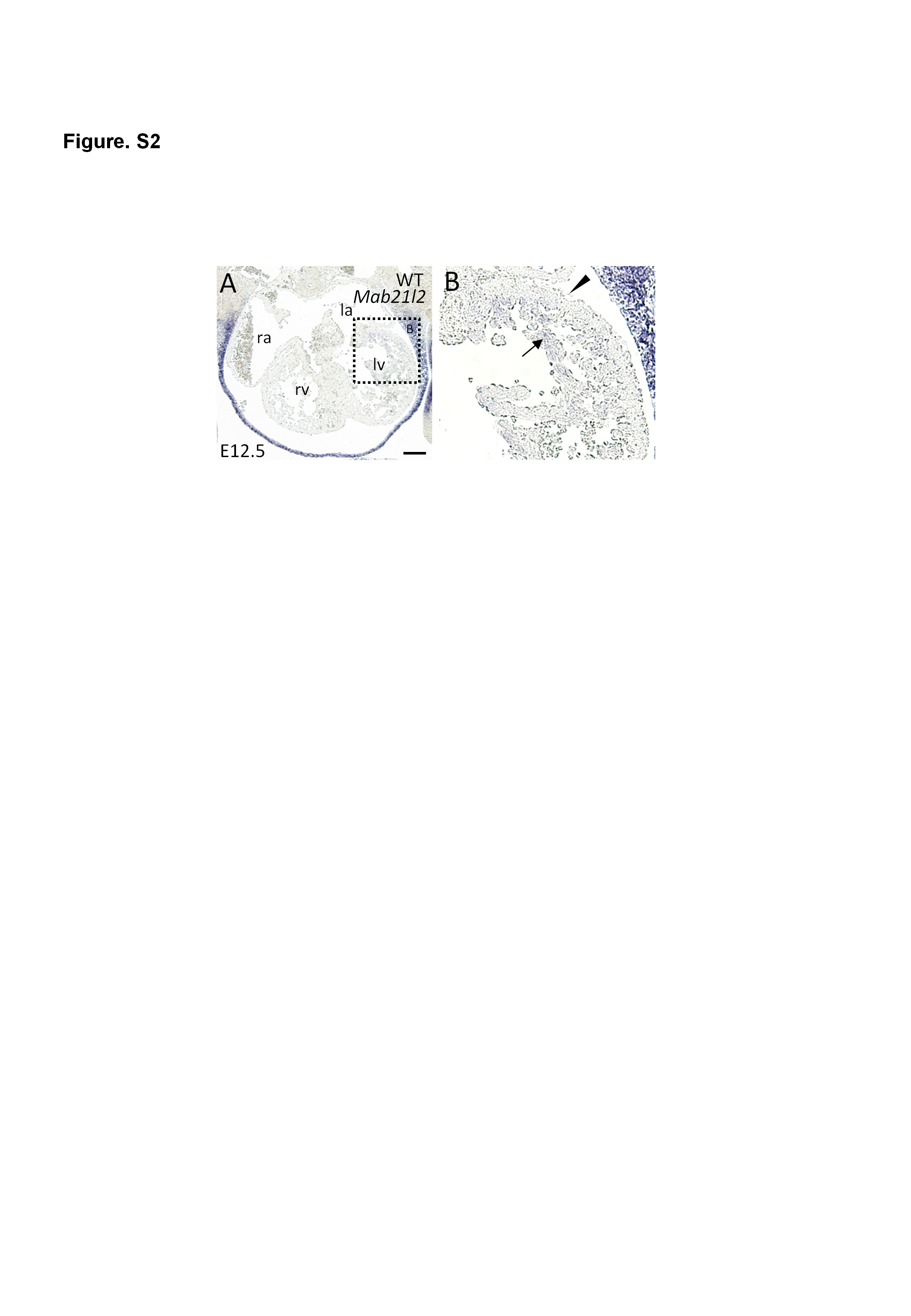

Supplement: Figure S2 — The expression of Mab21l2 at E12.5. (A and B) In situ hybridizations of transverse paraffin sections of E12.5 WT embryonic hearts for Mab21l2. Mab21l2 expression was detected at low levels in the trabecular myocardium (arrow), not in the epicardium (arrowhead) at E12.5 (A and B). la, left atrium; lv, left ventricle; ra, right atrium; rv, right ventricle. Scale bar represents 100 µm (A). (TIF) [file pone.0032991.s002.tif]

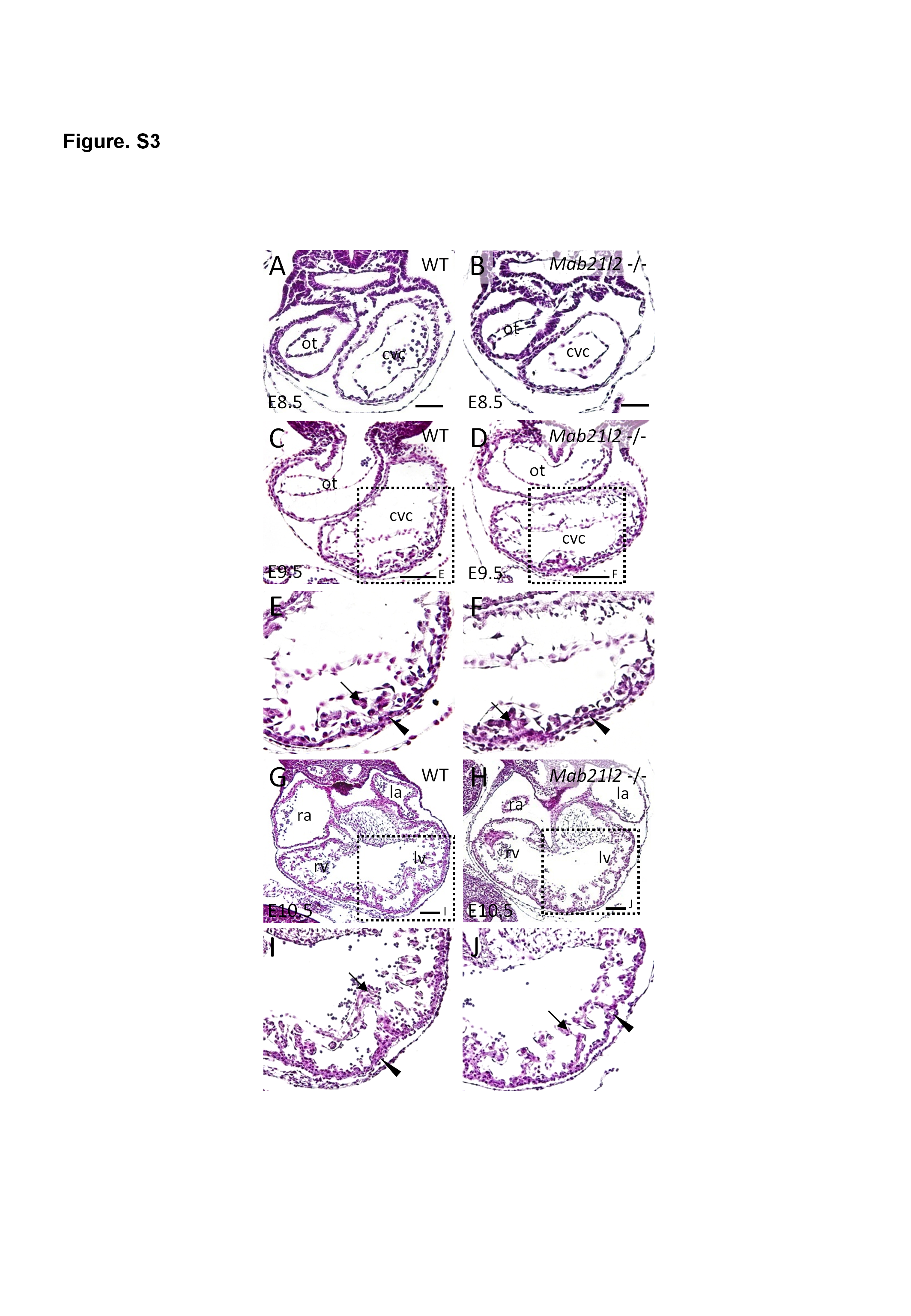

Supplement: Figure S3 — Defective morphogenesis in the heart region is observed from E10.5. (A–J) H&E-stained transverse sections of E8.5, E9.5 and E10.5 WT and Mab21l2 mutant embryonic hearts. Defects in the heart region were not observed in Mab21l2 mutant embryos (B, D and F) compared to WT embryos (A, C and E) at E8.5 (A and B) and E9.5 (C–F). However, thin compact myocardium was just visible in some Mab21l2 mutant (H and J) compared to WT embryos (G and I) at E10.5 (G–J). cvc, common ventricular chamber; la, left atrium; lv, left ventricle; ot, outflow tract; ra, right atrium; rv, right ventricle. arrows, trabecular myocardium; arrowheads, compact myocardium. Scale bar represents 30 µm (A and B), and 50 µm (C, D, G and H). (TIF) [file pone.0032991.s003.tif]

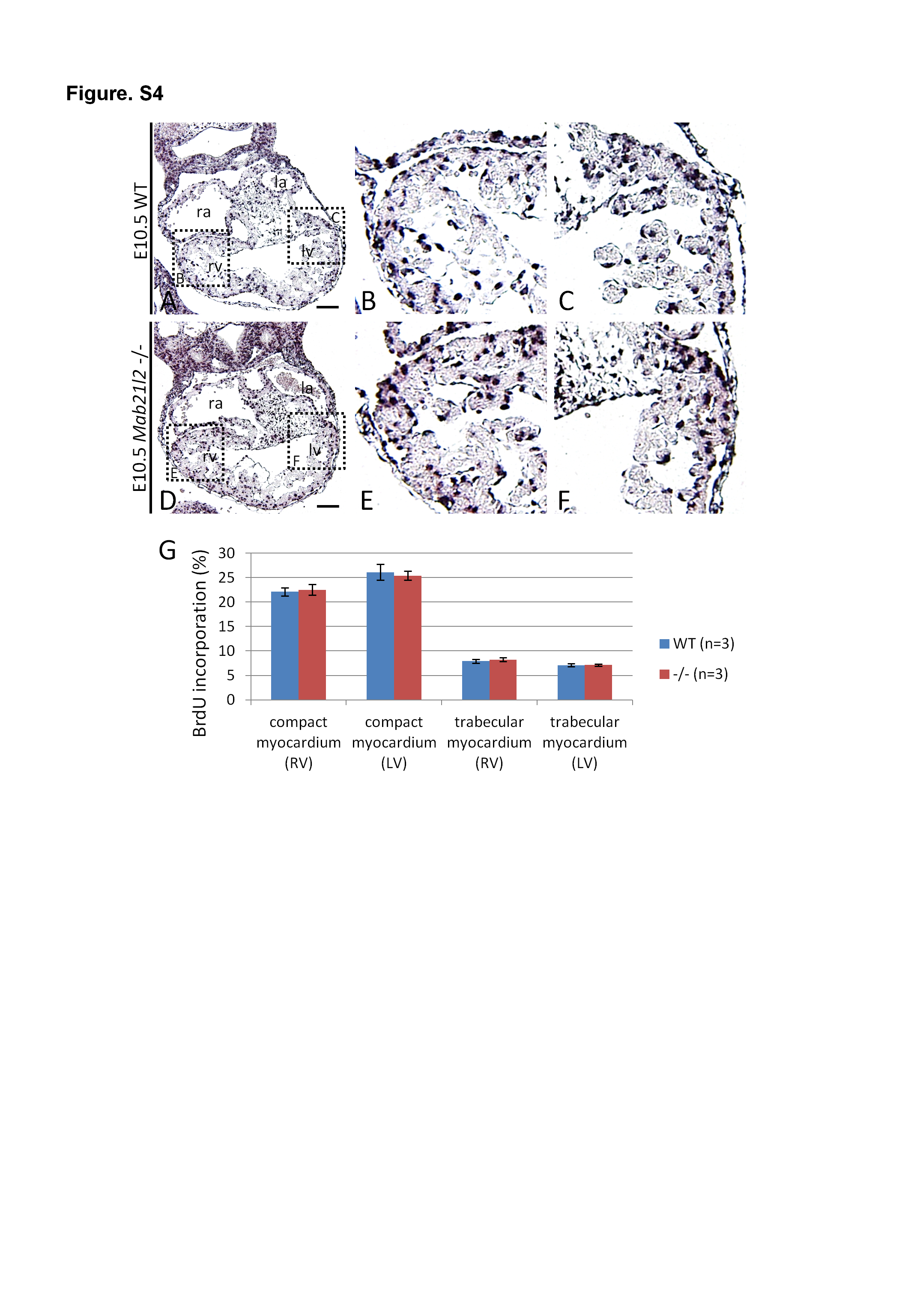

Supplement: Figure S4 — At E10.5, cell proliferation in the heart region of the Mab21l2 -mutant is unchanged compared to that of WT embryos. (A–F) BrdU assay on transverse paraffin sections of E10.5 WT and Mab21l2 mutant (−/−) embryos. BrdU staining shows that cell proliferation in the heart region of the Mab21l2-mutant (D–F) is normal, compared to WT embryos (A–C). (G) Quantification of BrdU incorporation. The percentage of BrdU-positive myocardial cells was calculated by dividing the number of BrdU-positive myocardial cells by that of the total myocardial cells identified by hematoxylin staining. The values show means of the proportions of BrdU-positive nuclei. Error bars represent the standard deviation. la, left atrium; lv, left ventricle; ra, right atrium; rv, right ventricle. Scale bar represents 50 µm. (TIF) [file pone.0032991.s004.tif]

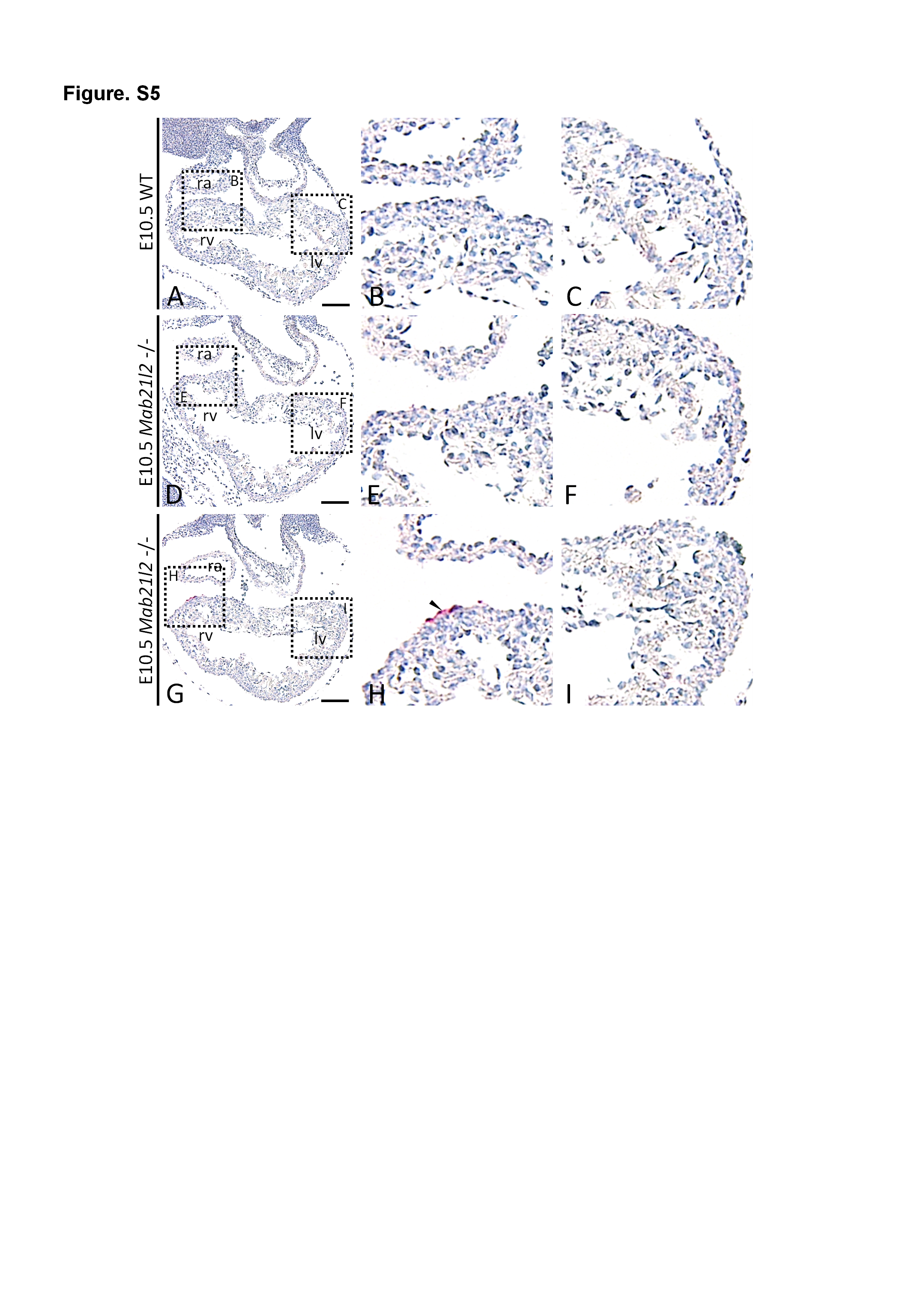

Supplement: Figure S5 — At E10.5, apoptosis in the heart region is generally unchanged in Mab21l2 -mutant compared to WT embryos. (A–I) TUNEL assay on transverse paraffin sections of E10.5 embryos. (D–F) In Mab21l2 –mutant embryos (D–F), apoptosis was not generally detected in the heart region, but in some Mab21l2-mutant embryos (G–I), increased apoptosis was observed. Arrowhead indicates TUNEL-positive cells (red). lv, left ventricle; ra, right atrium; rv, right ventricle. Scale bar represents 50 µm. (TIF) [file pone.0032991.s005.tif]

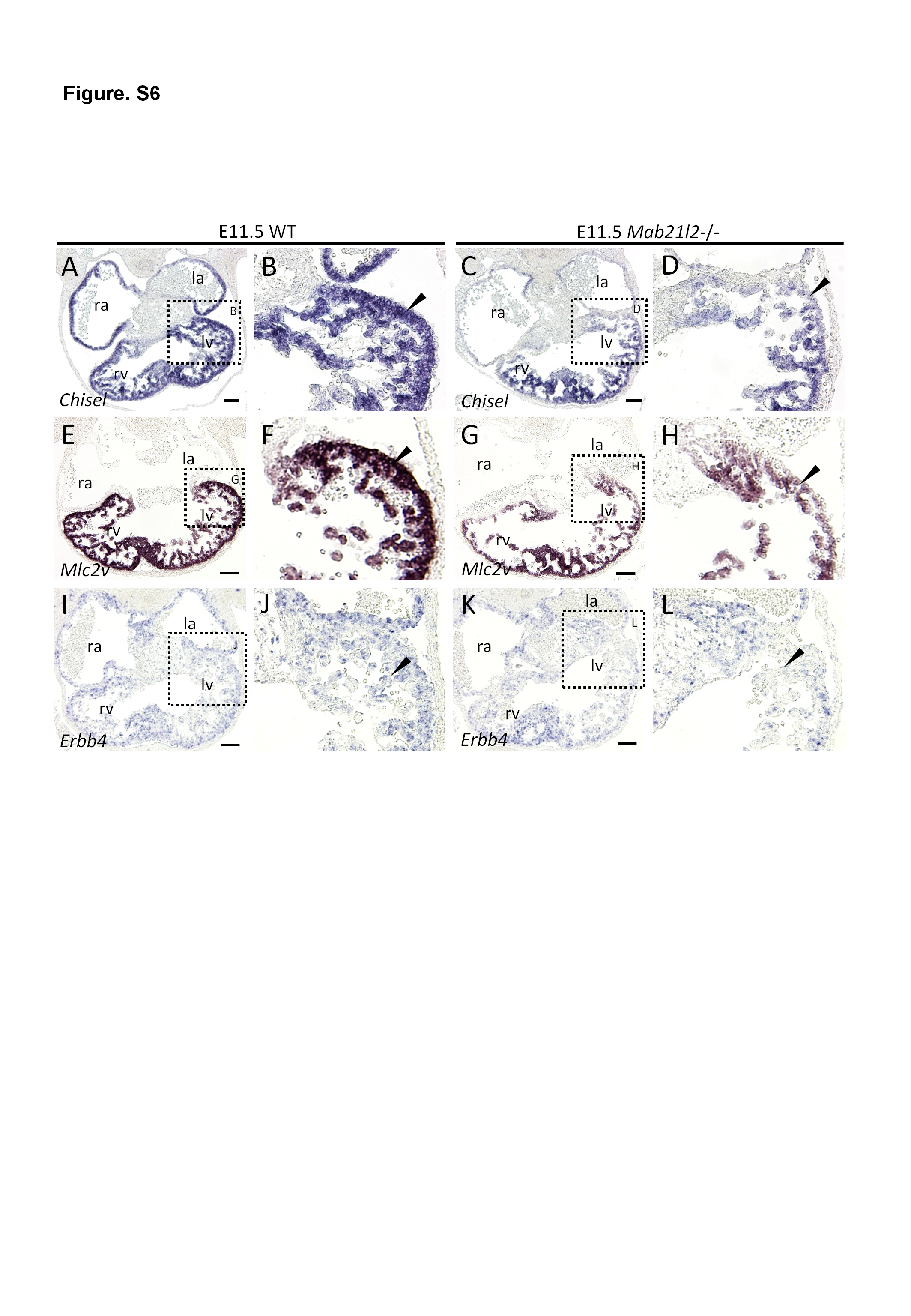

Supplement: Figure S6 — Mab21l2 mutants show defective expression of some myocardial differentiation markers and of Erbb4 essential for ventricular trabeculation. (A–L) In situ hybridizations of transverse paraffin sections of E11.5 WT and Mab21l2 mutant embryonic hearts for the transcripts indicated. The expression of Chisel and Mlc2v (cardiomyocyte differentiation markers), was reduced in the myocardium of Mab21l2 mutants (Chisel [C and D]; Mlc2v [G and H]; arrowheads) compared to WT embryos (Chisel [A and B]; Mlc2v [E and F]), especially in the dorsal region of the left ventricle (arrowheads). The expression of Erbb4 (essential for trabecular myocardial development in the heart ventricle) was also reduced in the Mab21l2 mutant myocardium (K and L) compared to WT embryos (I and J). la, left atrium; lv, left ventricle; ra, right atrium; rv, right ventricle. Scale bar represents 50 µm. (TIF) [file pone.0032991.s006.tif]

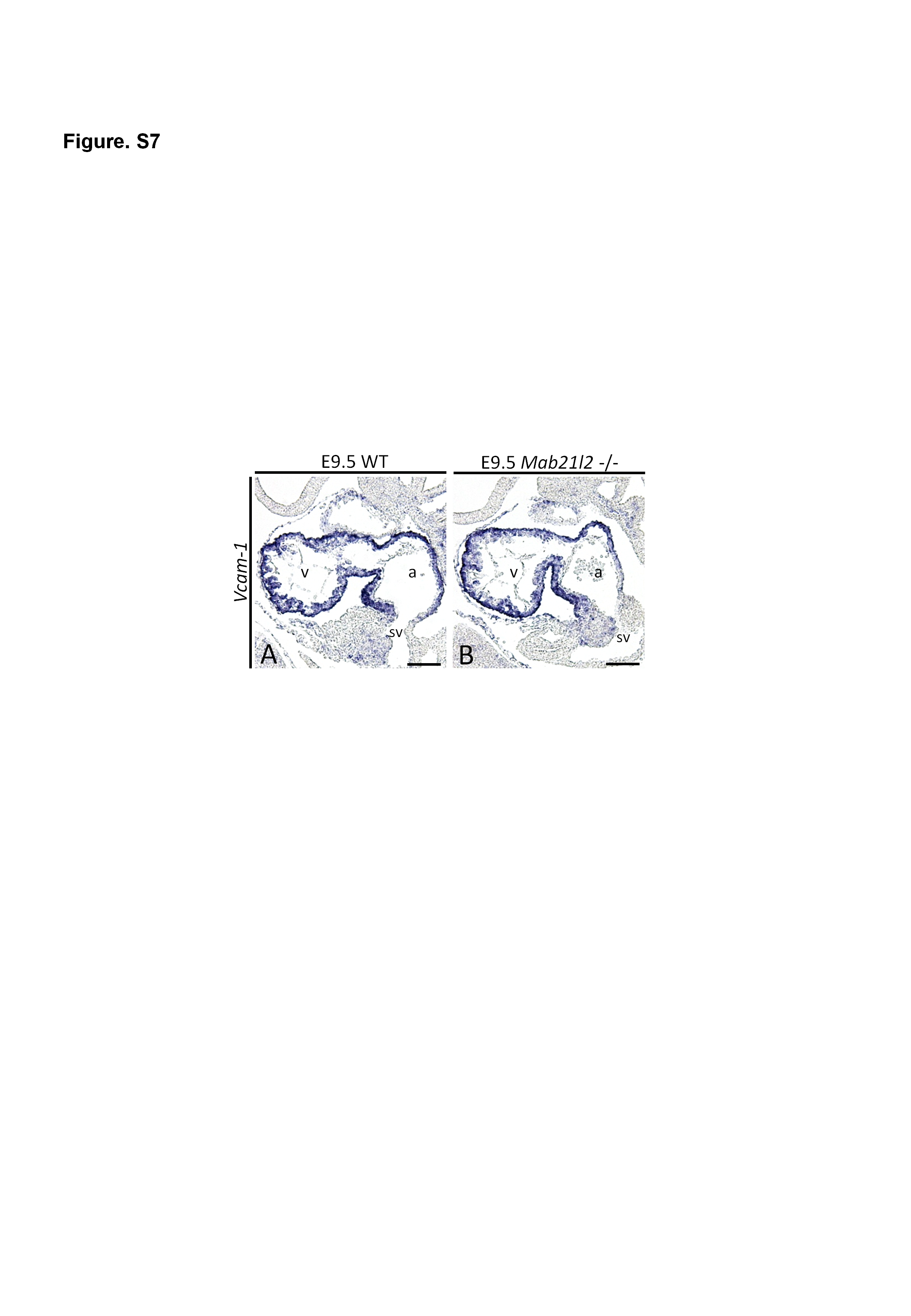

Supplement: Figure S7 — Vcam-1 expression was unchanged in Mab21l2 -mutant myocardium. (A and B) In situ hybridization analysis of Vcam-1 in sagital paraffin sections of E9.5 WT and Mab21l2 mutant embryos. (B) Vcam-1 was normally expressed in the Mab21l2-mutant embryo myocardium compared with WT embryos (A). a, atrium; v, ventricle; sv, sinus venosus. Scale bars represent 50 µm. (TIF) [file pone.0032991.s007.tif]

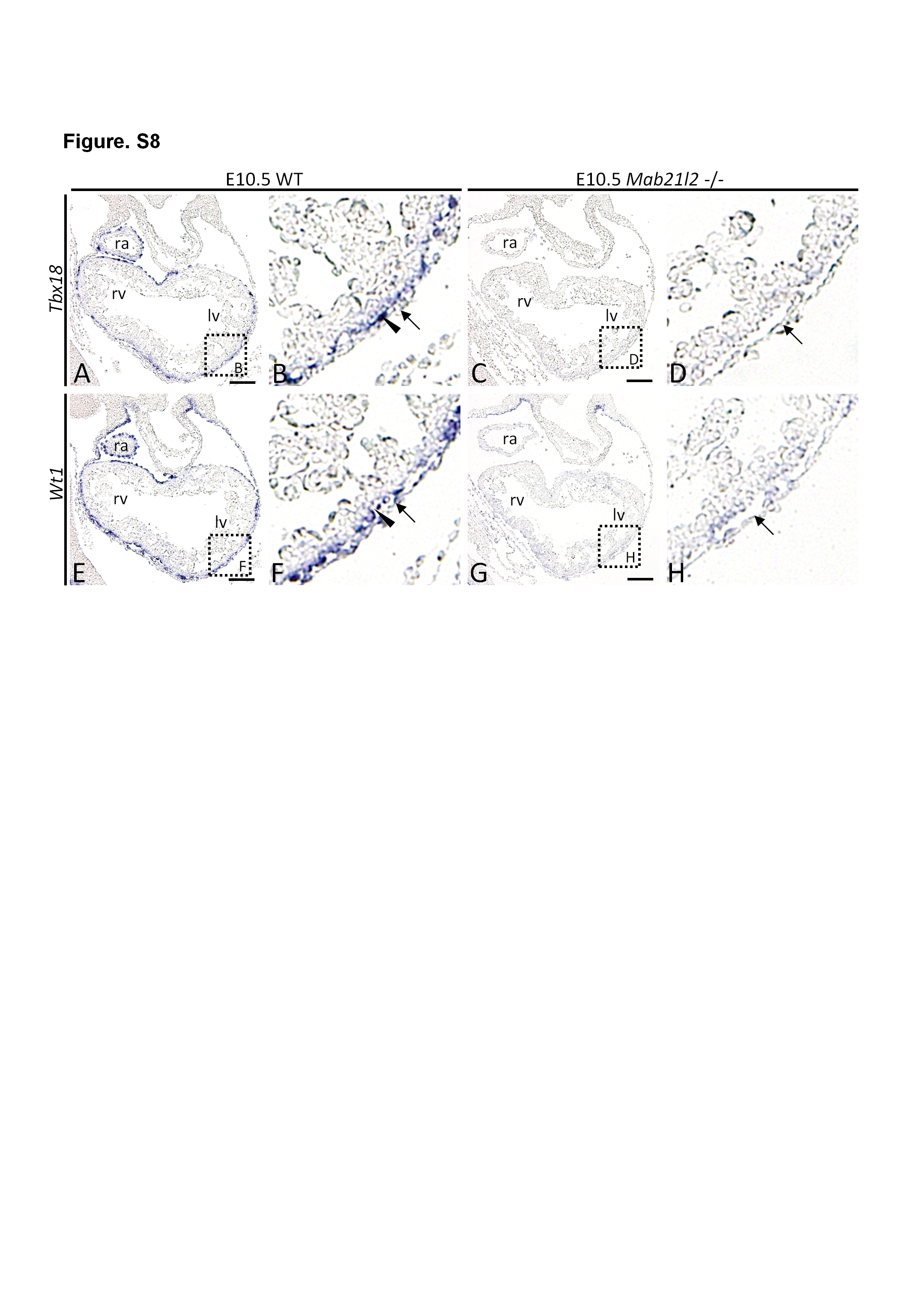

Supplement: Figure S8 — Defective morphogenesis of the epicardium occurred by E10.5. (A–H) In situ hybridizations of transverse paraffin sections of E10.5 WT and Mab21l2 mutant embryos for the transcripts indicated. The expression of epicardial marker genes was detected in E10.5 WT epicardium ([A and B] Tbx18; [E and F] Wt1), arrowheads), but not in Mab21l2 mutant embryos ([C and D] Tbx18; [G and H] Wt1) between the compact myocardium and the body wall (arrows). lv, left ventricle; ra, right atrium; rv, right ventricle. Scale bar represents 50 µm. (TIF) [file pone.0032991.s008.tif]

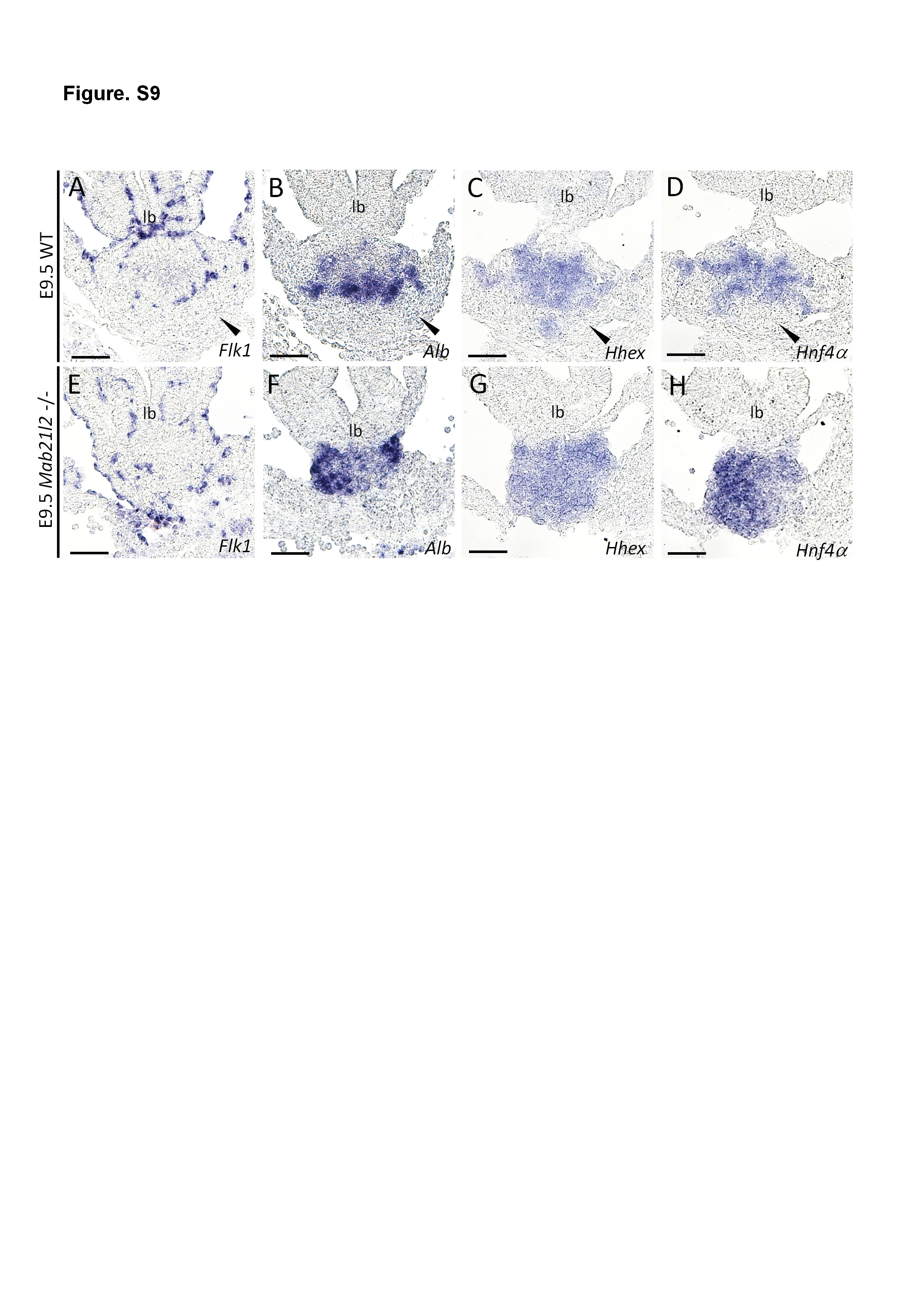

Supplement: Figure S9 — Defective morphogenesis of the STM does not affect the expression of the endothelial marker, Flk1 , or the hepatoblast markers, Alb , Hhex and Hnf4a . (A–H) In situ hybridization of transverse paraffin sections of E9.5 WT and Mab21l2 mutant embryos for the transcripts indicated. Flk1 expression was not altered in Mab21l2 mutant embryos (E), compared to WT embryos (A). The expression of the hepatoblast markers, Alb, Hhex and Hnf4α also remained unaltered in Mab21l2 mutant embryos (F–H), compared to WT embryos (B–D). lb, liver bud; arrowheads, septum transversum mesenchyme. Scale bar represents 30 µm. (TIF) [file pone.0032991.s009.tif]
